# City Data Hub: Implementation of Standard-Based Smart City Data Platform for Interoperability

**DOI:** 10.3390/s20237000

**Published:** 2020-12-07

**Authors:** Seungmyeong Jeong, Seongyun Kim, Jaeho Kim

**Affiliations:** 1 Autonomous IoT Research Center, Korea Electronics Technology Institute, Seongnam 13509, Korea; sm.jeong@keti.re.kr (S.J.); seongyun.kim@keti.re.kr (S.K.); 2 Department of Electrical Engineering, Sejong University, Seoul 05006, Korea

**Keywords:** smart city, data platform, data hub, data marketplace, NGSI-LD, oneM2M

## Abstract

Like what happened to the Internet of Things (IoT), smart cities have become abundant in our lives as well. One of the smart city definitions commonly used is that smart cities solve city problems to enhance citizens’ life quality and make cities sustainable. From the perspective of information and communication technologies (ICT), we think this can be done by collecting and analyzing data to generate insights. The City Data Hub, which is a standard-based city data platform that has been developed, and a couple of problem-solving examples have been demonstrated. The key elements for smart city platforms have been chosen and they have been included in the core architecture principles and implemented as a platform. It has been proven that standard application programming interfaces (APIs) and common data models with data marketplaces, which are the keys, increase interoperability and guarantee ecosystem extensibility.

## 1. Introduction

Smart cities are everywhere these days. Like we have been hearing for the Internet of Things (IoT) from TV commercials, such as smart home services, even on load signs there are big sign boards promoting smart cities and their solutions.

Since smart city is not a technical terminology but rather a concept, there are a lot of definitions for a smart city from standard development organizations, research papers and also private corporates [1]. The one commonality of those definitions is that a smart city enhances citizen life quality by solving city problems. There should be many non-technical aspects to achieve them, but from the perspective of information and communication technologies (ICT), data can help in realizing the smart city mission. To do so, smart city systems, whether it currently existing or newly deployed later, should be interoperable in terms of data sharing and utilization.

Among the many technical keywords that comprise smart cities, for instance, IoT and big data, analytics fits well to solve city problems. In IoT-enabled smart cities, IoT systems send real-time data from city infrastructures having thousands of sensor devices to smart city data platforms, while legacy systems provide relatively static data (e.g., statistics files). Osman, A.M.S. [2] summarizes several research studies on big data analytics use cases for smart cities, such as for traffic control and crime analysis. There, we can find needs for a smart city data platform having big-data-handling capabilities. As happened to IoT, in the market and research projects, new solutions are being launched for different application domains and, in many cases, those are silo solutions. When solutions are fragmented without interoperability, achieving economies of scale gets harder. Cities become less interoperable and fall into vendor lock-in. Furthermore, it becomes harder to re-use the tons of data collected due to expensive processing price.

Several core concepts regarding smart city interoperability have been studied and defined. The National Institute of Standards and Technology (NIST) conceptualized the pivotal point of interoperability (PPI) in an IoT-Enabled Smart City (IES-City) framework for smart cities analyzing IoT platforms including oneM2M standards [3]. The Open and Agile Smart Cities (OASC) initiative defines minimal interoperable mechanisms (MIMs), those are standard application programming interfaces (APIs), data models and data marketplaces, and implemented them in the H2020 SynchroniCity project as a large-scale smart city pilot project [4]. These common interoperability-assuring topics became the essence of the smart city data platform, called the City Data Hub, which is illustrated in this paper.

From 2018, two ministries in South Korea, the Ministry of Land, Infrastructure and Transport (MOLIT) and the Ministry of Science and ICT (MSIT), jointly launched the National Strategic Smart City Program. With over 100 consortium members, it has one R&D project which mainly delivers the City Data Hub and two other projects for test-bed pilots in Daegu and Siheung cities [5]. The reason to have this structure is to develop a data-driven smart city platform and prove it in the two cities. With that proof, the goal is to exploit the platform and provide other solutions to other cities in Korea and also abroad.

The main contribution of this work is that our result, the City Data Hub, realized the MIMs while solving smart city interoperability issues, especially data-level interoperability. In our case, we leveraged the Next Generation Service Interfaces-Linked Data (NGSI-LD) standards. Interoperability of the interfaces and data played a pivotal role in designing an architecture having different functional modules. With this modular architecture, new functional modules complying with the NGSI-LD standards can be added later. Furthermore, data distributed on the data marketplace should be understandable by third-party applications.

The remainder of this paper includes the following topics. Section 2 provides a summary of the related works on data interoperability, standard APIs, data models and data marketplaces pertaining to smart cities. Section 3 defines the main features of the City Data Hub and Section 4 illustrates modules of the City Data Hub to see how they are interconnected with the NGSI-LD standards. In Section 5, two proof-of-concept examples are demonstrated to show how the domain data, with interoperability, in the smart city data platform are used by smart city applications. Lastly, in Section 6 and Section 7, we summarize the remaining research areas and conclude our work.

## 2. Related Works

### 2.1. Interoperability Levels

Interoperability for data exchange in general, which can also be applied to smart cities, includes interface, syntactic and semantic levels [6]. Interface-level interoperability can be achieved by common APIs. To meet data-level interoperability, syntactic-level and semantic-level interoperability also needs to be satisfied. Syntactic-level interoperability defines the format and data structure, so this can be met by protocol specifications which normally come with interface specifications for system design. Lastly, semantic-level interoperability can be guaranteed with common data models or ontologies. When data are exchanged among different smart city applications, for instance, the meaning of the data is correctly understood.

In this paper, we use standard APIs and common data models for data-level interoperability. Since different stakeholders provide and consume data on smart city data marketplaces, the data interoperability should be guaranteed. When it comes to the smart city, data interoperability breaks the data silos in heterogeneous vertical domains and fosters new business opportunities.

### 2.2. Standard IoT APIs and Data Models

As an IoT standard, oneM2M defines its architecture and APIs for commonly required capabilities of IoT applications in different domains [7]. Like in smart cities, IoT applications can be categorized in many domains, such as home, energy, vehicle, environment, etc. Instead of vertical silos, oneM2M delivers a horizontal common platform with middleware functions, including data sharing. Busan, Goyang and Daegu deployed oneM2M standard-compatible platforms to seek interoperability and lower the development time and cost [8].

Since the Release 2 of oneM2M, after the initial standard publication, data models have also been standardized for interoperability. Starting from home domain appliances, now, models for industry domains are being defined [9].

The other IoT standard, Open Connectivity Foundation (OCF), also specifies device models and standard APIs for vendors’ interoperability, starting from smart homes [10,11]. Later, there was the collaborative work between oneM2M and OCF to harmonize device models in a common area, which is the smart home. Since each standard data model follows its representational state transfer (REST) resource structure and data types in its protocols, the models cannot be simply unified but can be harmonized in terms of information models. After the collaboration, oneM2M reflected this harmonization in the Release 3 specification and OCF published the new one from this effort [12].

In smart cities, the FIWARE foundation published some domain models for its NGSI-based IoT platforms [13]. After FIWARE, the European SynchroniCity project, referring to the FIWARE data models, added new ones for its pilot services [14].

In our work, we use the standard APIs for interface- and protocol-level interoperability. We also use common data models to provide data-level interoperability among smart city applications, as well as functional modules of the City Data Hub.

### 2.3. NGSI-LD

Extending OMA (Open Mobile Alliance) NGSI-9/10 interfaces [15] for linked data, ETSI Industry Specification Group (ISG) Context Information Management (CIM) published the NGSI-LD APIs [16] with its information model scheme [17]. As the extension to the OMA interfaces, which were used for IoT open-source platform implementations in FIWARE (e.g., Orion Broker), IoT systems provide measurement data to NGSI-LD systems. As aforementioned, a oneM2M system is also one data source for the NGSI-LD system. In this paper, in the context of an IoT-enabled smart city, we have implemented a oneM2M adaptor to collect IoT data from oneM2M platform.

**Figure 1:**
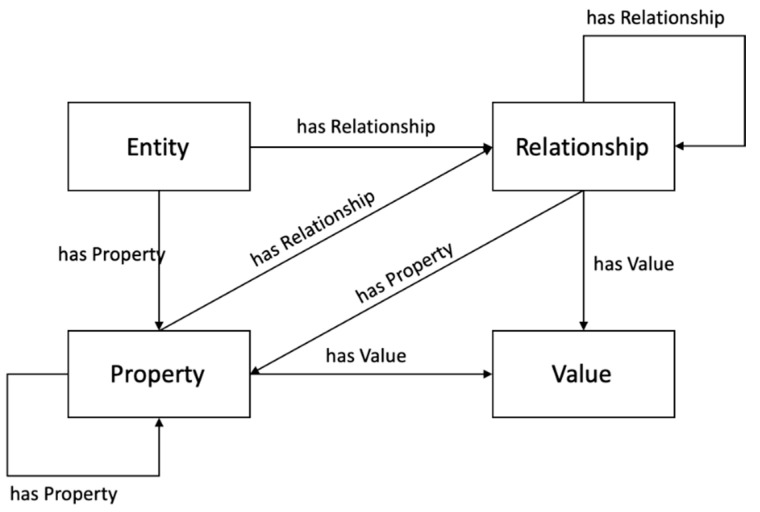
Next Generation Service Interfaces-Linked Data (NGSI-LD) Information Model.

Compared to the former OMA NGSI, the information model grounding of the NGSI-LD, as the name says, incorporates the linked data concept and is based on graphs; hence, it lets developers focus more on information with relations [18]. Figure 1 illustrates the NGSI-LD information model [17] which can express real-world entities with properties and relationships. With the scheme, even properties of a property or properties of a relationship can be modeled. Having a relationship concept for linked data entities, the NGSI-LD information model scheme can be useful for interconnecting and federating other existing information models [19,20]. For smart city use cases that deal with heterogenous data, different models can be mashed-up, especially for cross-cutting-use cases, with the NGSI-LD information scheme.

NGSI-LD specifies RESTful API, which is a web-developer-friendly interface style on top of the NGSI-LD core concept. In addition to the previous NGSI interfaces, the NGSI-LD API defines historical data retrieval APIs.

As the adoption example for the NGSI-LD API and its information-model-compatible data models, [18] showed that its standard interfaces helped the integration of different device vendors in the agriculture domain. NGSI-LD also has been used in other domains, such as smart water [21] and aquaculture [19], for example. In case of SynchroniCity, some of the atomic services in smart city domains also implemented NGSI-LD [22].

In our proof-of-concepts, we show how the NGSI-LD standard interfaces and compatible data models provide interoperability through the data flow, which is from acquiring external system data to sharing processed and predicted data to the applications.

### 2.4. Data Marketplaces

As a horizontal data platform for smart cities, data distribution among various data stakeholders in heterogeneous service domains can be much more activated by data marketplace enablers. A data marketplace enables data producers to sell and provide their data while consumers find interesting datasets and then purchase or use them.

Open data portals used to offer static data (e.g., statistics and logs) which were updated seldomly and manually in file formats. Data portals combined with IoT can provide live data with the source platform APIs. For instance, oneM2M-compliant platforms in Korean smart cities run open data portals based on oneM2M standard APIs [23,24].

The SynchroniCity project developed a data marketplace based on Business APIs from TM Forum (TeleManagement Forum) and its implementation Business API Ecosystem (BAE) Generic Enabler (GE) from FIWARE [25]. The work has demonstrated a marketplace with service-level agreement (SLA) and license management features, for example, that are additional capabilities to the BAE GE.

Noting that the oneM2M marketplace deploys heterogeneous marketplace enablers on its own web services, in our approach, which is similar to FIWARE, dataset management is done by the platform which can be interworked with different marketplace portals. In our work, we implemented the data marketplace enabler as the internal functional module of the City Data Hub, which is a different approach from FIWARE. The internal module holds necessary information for datasets while leaving original data to the Data Core module. Integrated authorization is also provided for users from data ingestion to dataset management.

## 3. City Data Hub Architecture

### 3.1. Introduction

To achieve data-level interoperability on the City Data Hub, as summarized in Section 2, we endorsed NGSI-LD interfaces and defined NGSI-LD-compliant data models. From data ingestion, the data in common data models flow between the data handling modules over the standard APIs. When prepared as datasets by the marketplace enabler module, smart city applications receive datasets that are in the NGSI-LD format.

With the three pillars (i.e., standard APIs, data models and marketplaces) for interoperability, the following design principles for the City Data Hub architecture were derived.

- Interoperability with standards

The data-storing module shall adopt standard APIs with pre-agreed data models among interested parties. Then, it can interwork with other modules in the City Data Hub and external services or infrastructures.

- Maintainability with openness

Each module should be easily replaceable by other implementations. The technical specifications for its interfaces and protocols should be published. Then, the platform can be evolved with open participation and avoid vendor lock-in in terms of specifications and implementations.

- Extensibility with ecosystem

While several features are natively supported (e.g., cloud management and security), a new common functional module can be contributed. Then, the interoperability mechanism above ensures that the new module can be easily integrated. This can establish the City Data Hub ecosystem for other organizations as well as the original project members.

### 3.2. Architecture Overview

**Figure 2:**
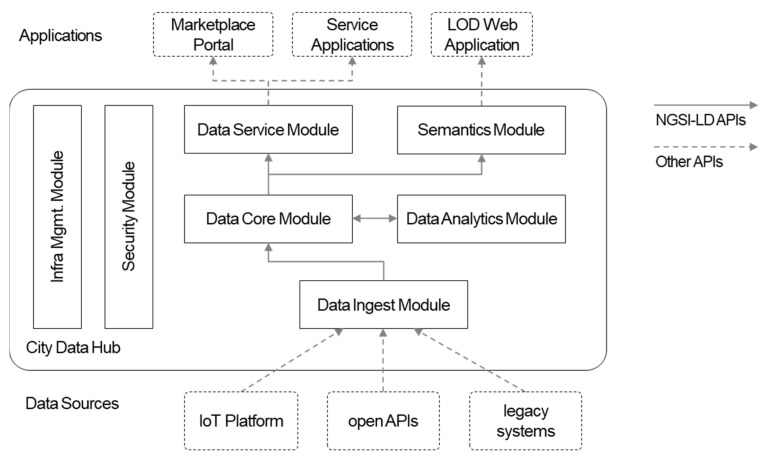
City Data Hub high-level architecture with data flows.

The City Data Hub, which has high-level architecture with data flows, as depicted in Figure 2, follows modular architecture so that several modules can be deployed and new modules can be integrated later. Data are stored and shared in the data core module. The other modules involved in data management (e.g., ingestion and service), putting the data core in the center, face each other with the standard interfaces of the data core.

The NGSI-LD APIs are applied to the data core module, and the data flows in the City Data Hub go through the standard interfaces. Data from external sources over their proprietary APIs are collected and sent to the data core module. At this point, converted data in the common data model are stored using the NGSI-LD APIs. The analytics module receives data from the core, and outputs, such as prediction results, can be also stored in the data core. The data in the core module can be served by the data service module and the service module receives data via the standard APIs of the data core module. When data are finally received by service applications, the data are represented in datasets, which are the array of NGSI-LD entities in NGSI-LD format.

The data ingest module is responsible for ingesting city data into the data core. City data from different systems, platforms or infrastructures are heterogeneous in terms of their APIs and data models. Adaptors in the ingest module receive data from the source with their APIs, translate data models and ingest data using the core module’s APIs. As a framework, users can define adaptors to be able to interwork with proprietary solutions.

Ingested data are stored in the data core module. The core module supports NGSI-LD APIs and compatible data models. When the core receives data from the ingest, data model validation is performed so that it can provide data that are compatible with the given data models. The data, historical as well as latest, are served to other modules with the standard APIs.

The data service module is the consumer of the core module APIs and serves as the back-end API server for the smart city data marketplace portal. On the data marketplace, users can sell or purchase data that are contained in the City Data Hub. To smart city applications, which are located outside the City Data Hub, city data are provided as datasets. A dataset is a set of data endpoints (i.e., an entity instance in NGSI-LD API) and is created by data providers who have ownership of data. When the dataset is purchased on the marketplace portal, as the authorization mechanism in the City Data Hub, the user who made the purchase with the credential can access the dataset on their applications.

Data analytics is also one of the major functionalities of the City Data Hub. Benefits of having data analytics as part of the data hub are that (1) domain applications do not need to build their analytics servers for common preprocessing, model training, testing and deployment; (2) mash-up (e.g., weather and parking) is done in analysis and prediction since different data are being collected at the platform; (3) analytics results can also be served to others.

Data scientists can create analytics models on their sandboxes, having some sample data from the ingest module. To build models, a set of algorithms and data preprocessing functions are supported. A model is trained and tested so it can be requested for the analytics admin to deploy it on the data hub. When it is deployed, ingested data are provided to the model and then the generated prediction output is stored into the data core.

Semantic data (i.e., Resource Description Framework (RDF) triples) are another form of data served by the semantics module. Users can perform SPARQL Protocol and RDF Query Language (SPARQL) queries on the semantic data to obtain semantic knowledge, for example. The module semantically annotates data with smart city ontologies. Depending on policies, raw data can be given by the ingest module or the core module with NGSI-LD APIs.

Authentication and authorization are handled by the security module and the API gateway. The infra module is responsible for cloud infrastructure management for the other modules, including resource monitoring, auto scaling, etc., for hybrid (i.e., public and private) cloud infrastructures.

## 4. City Data Hub Modules

### 4.1. Data Ingest

The data ingest module mainly provides two features: (1) ingest adaptor management and (2) API and data model conversion. The ingest adaptor is an interworking proxy between external data sources and the data core module. Therefore, the main role of the adaptor is to adopt data from the source interface, convert data and ingest to the core. The interfaces of those external data source systems are different from the NGSI-LD APIs of the data core. Furthermore, data models are different in terms of data properties’ semantics and syntax. This is the reason why the ingest module supports building adaptors with data model conversion templates by users (e.g., ingest module managers).

Apache Flume [26] has been used to implement the ingest module, so its core concept to manage adaptors is similar to that. Whereas each adaptor collects, converts and ingests data per external data source, an agent is a logical group of adaptors. From a management perspective, an ingest manager can create an agent to manage a set of adaptors for the same protocols, geographical region, etc.

For well-known IoT standard interfaces and protocols, several pre-defined adaptors are provided. For example, data from oneM2M standard protocols [27] can be easily ingested with target platform-specific configurations since oneM2M adaptor is supported by default. Those adaptors can be re-used in different cities that already have platforms with the standard APIs. In South Korea, oneM2M interfaces have been deployed in smart city pilot projects since 2015, and now, commercial services are being offered in other municipalities [8].

In case of oneM2M APIs, subscription/notification APIs are supported by the adaptor. The adaptor setting includes discovery target in a oneM2M resource tree. A list of data sharing resources (e.g., container), which will be ingested into the data core, can be selected during the set-up. When the user selects a number of resources from the discovery result, subscriptions are made by the adaptor so that it can receive notifications for new data events (e.g., new contentInstance creation).

When new data are delivered by an asynchronous notification reception or a data polling response, the adaptor performs data model conversion per pre-configured conversion rules. In the data model, each entity property specifies a data type. For example, the ‘OffStreetParking’ entity type has the ‘availableSpotNumber’ property with integer data type.

As an adaptor, when a data message is received, it is not easy to determine whether it should use the NGSI-LD entity create function or update request. This is because keeping all entity instance status information with identifier mapping between a source and NGSI-LD entity is difficult. As a compromise, in the data hub, the adaptors perform an upsert operation, meaning they try to update first, and if that fails, they send the create request.

### 4.2. Data Core Module

#### 4.2.1. Standard API Implementations

Within the City Data Hub, the data core module realizes NGSI-LD centralized architecture as the central context broker, while the ingest module acts as the context producer. The core module implements context information provision, consumption and subscription APIs in the NGSI-LD specification [16].

Context information provision APIs are used by the ingest module to create new NGSI-LD entity instances and to update the instances. In addition to HTTP (HyperText Transfer Protocol) binding, as specified in the standard, Kafka [28] binding is also applied for provision APIs.

**Figure 3:**
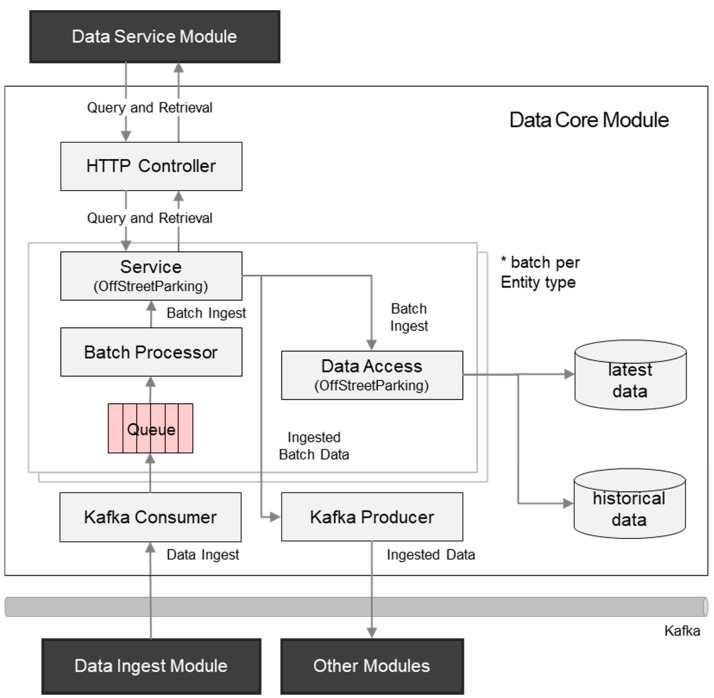
NGSI-LD application programming interface (API) with HTTP and Kafka binding.

Kafka is one of the message queue solutions, and message queues are preferred when a gap between a source and a destination system is big enough. Furthermore, when a data flow is not consistent and, sometimes, a large volume of data is sent unexpectedly, a message queue is the better choice than connection-oriented protocols between the source and the destination. While HTTP can deliver a request at once, a Kafka consumer can receive multiple messages together. This is not the same as the batch operation in NGSI-LD since the batch request is determined by the client, so the server has no choice. On the other hand, in Kafka, the consumer can decide when and whether to consume one or more messages depending on the system status. Lastly, normally, an API server’s performance is limited by a database management system (DBMS), and maximum server performance can be achieved by leveraging the DBMS bulk operation. This can be done using Kafka message queue management. Figure 3 describes how Kafka is implemented to achieve higher performance.

After the context provision, the entity instances are consumed by other modules, such as the data service with discovery and retrieval APIs. As the specification defines, a consumer can obtain the latest status of the entity with the retrieve entity API and historical data with the query temporal evolution of entities API. In the City Data Hub implementation, historical data are not explicitly ingested with the corresponding resource ‘/temporal/entities’ and their sub-resource APIs. Instead, new instance fragments with a timestamps property (e.g., observedAt) are stored as a historical database. This is also depicted in Figure 3. A data ingest request over Kafka is received and reflected on both databases.

As the central broker that implements both APIs, when there is an entity data provision to the entity resource, the data core provides historical data to consumers when GET ‘/temporal/entities’ API is requested.

For event data consumers, for example, the semantic annotator in the semantics module or the notification handler in the data service module, the NGSI-LD subscription/notification feature provides event push, so near-real-time data consumption becomes possible.

#### 4.2.2. Access Control

Access control basically defines who can access what—e.g., access control policy handling procedures in oneM2M [29]. ‘Who’ (e.g., Application Entity) is defined in the oneM2M architecture as logical entities, which send requests and responses, and there are several access control mechanisms defined. Unlikely, NGSI-LD only specifies APIs, without defining logical entities that initiate the APIs, so there is no such concept specified yet pertaining to authorization. In the OMA NGSI, this was the same, so external authentication and authorization as well as identifier management components were integrated to build FIWARE-based IoT platforms [30,31,32].

When it comes to the City Data Hub, so far, the NGSI-LD APIs are not exposed to external applications directly. Rather, the applications can receive data in the core via the data service module. When a dataset is purchased by a user, the user acquires access rights to retrieve the set of data instances. Section 4.3 illustrates further details.

Later, when the data core interfaces need to be accessed directly by external applications, access control policy configurations and decisions can be supported by the security module per NGSI-LD resources (e.g., /entities/{entity}).

#### 4.2.3. NGSI-LD Data Modeling

To use NGSI-LD APIs in smart city services, data modeling needs to be defined to implement the APIs. We have made two proof-of-concept (PoC) services and reached the following principles [33].

##### NGSI-LD Entity Modeling Principles

- A model per entity

The target for modeling is a logical entity, such as a parking lot. There may be different perspectives on the same thing; however, the model we define binds to a given smart city service, for example, a parking service. Therefore, other aspects such as building size or price in real-estate service could be defined but as a different entity type.

The opposite concept to the logical entity is a physical entity. We took an approaching using services, not devices, that generate sensing data to define data used in applications so that entities represent logical things, such as weather forecasts. In the case of OffStreetParking and ParkingSpot, which seem to represent physical concepts, in Section 5.1, they includes logical aspects, such as availableSpotNumber.

Still, physical representation as a type is possible. When there is an air quality sensor deployed downtown, each sensor can be modeled to provide the geo-location and manufacturer information as well as its sensor reading, if needed.

- Raw data vs. processed data

When raw data from various data sources are stored in the City Data Hub, some of them are used for mash-up and prediction. From our experience, it is better to distinguish raw data and processed data at the entity level. In other words, we prefer not to mix processed data properties with raw data properties in one entity type. A possible downside of mixing those two up is that license and ownership resolutions become difficult.

- Linkage between processed data and raw data

If having separate model definitions for the raw and processed data, there should be a link between the two so that applications can obtain the other by reference.

#### 4.2.4. Dynamic Schema for New Data Models

As the data core, for API implementation, we selected PostgreSQL which is the powerful Relational Database Management System (RDBMS) with richer geographical query features than other database types. Still, whenever a new data inflow needs to be provided to the City Data Hub, developing NGSI-LD API sets for each data model could be really demanding. Therefore, a dynamic schema API for NGSI-LD modeling has been defined. Adding a new schema can be performed by creating an API and it is translated into the corresponding data definition language (DDL) in the database to create a new database table.

The main motivation to define a new schema description notation, rather than using the JSON (JavaScript Object Notation) schema [34], is to support NGSI-LD-specific features and have a better performance-related property. The pre-defined attribute types, such as Property and Relationship with their data structure following the JSON-LD [35], are supported. Furthermore, more concise notation than the JSON schema makes modeling and implementation easier for modelers and developers, especially for data models that have nested structures (e.g., Property of Property). Note that this is allowed in the NGSI-LD information model [17]. This structure makes it difficult for developers to write API codes as well as to define new entity types with the standard schema. With regard to the performance, the index columns (i.e., indexAttributeIds) for an RDBMS implementation can be optionally set to have a higher transaction per second (TPS). Table 1 shows the overall schema structure.

To represent the nested attribute structure (e.g., Property of Property), the Attribute complex data type is defined. When a Property value has several data elements (e.g., address having country code, street address, etc.), a JSON object can be specified. Table 2 specifies child properties of the Attribute data type.

Table 3 defines the ObjectKey data type, which is also the means of structured data representation.

**Figure 4:**
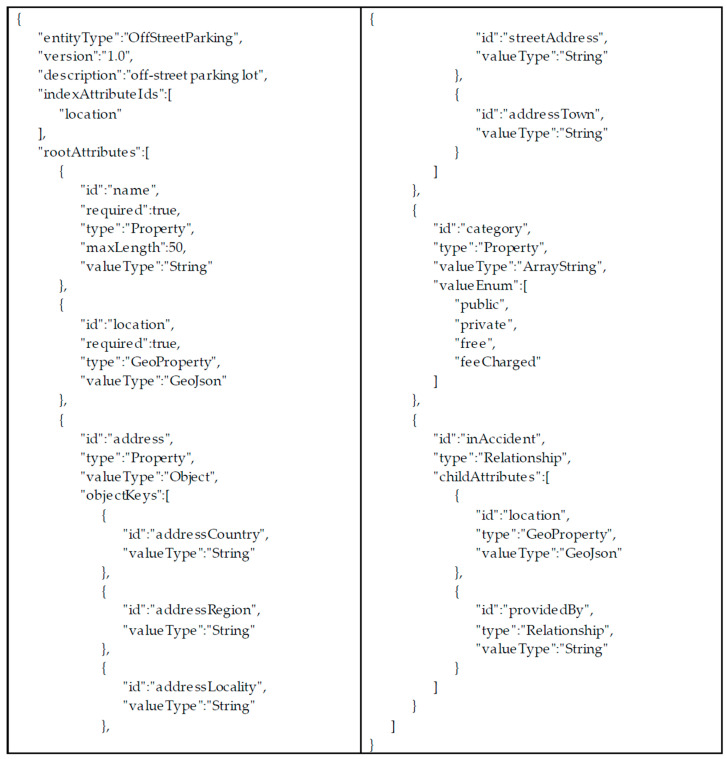
Schema definition example of an off-street parking lot.

Figure 4 provides the example of OffStreetParking entity schema definition with the above notation. With this NGSI-LD modeling-friendly notation, the data core module can easily adopt new model definitions with minimum implementation overhead.

### 4.3. Data Service Module

Level 4 of the SynchroniCity framework suggests deploying a data marketplace which distributes city data among different data providers and consumers [4]. The data service module in the City Data Hub plays the back-end API server for the data marketplace portal. When the provider registers a new dataset to the data marketplace (i.e., a dataset creates an API), the portal admin grants it, so then, the consumer requests to use the dataset with the corresponding API. While defining a dataset, the provider can modify attribute names given in the NGSI-LD data models from the core into a preferred one. There is also the option to choose whether the user retrieves the dataset later or receives event notifications which originate from the data core. For both cases, pre-defined attribute name mappings in the dataset configuration are applied to the dataset instances contained in the dataset responses and notifications to the applications.

The aforementioned dataset groups a list of existing NGSI-LD entity instances. For entity instances that can be added or deleted later, a filter-based dataset can also be defined. Whenever dataset retrieval is requested by an application, the data service module receives instances from the core with the entities query API with the given instance filter.

**Figure 5:**
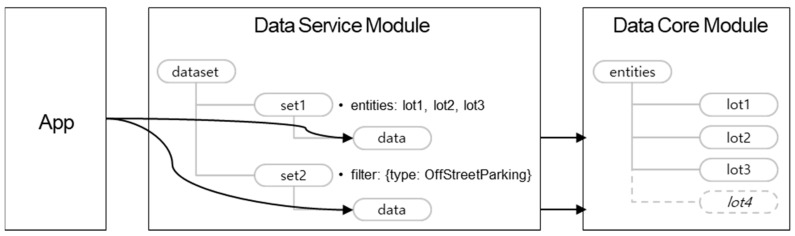
Dataset management with data core.

In Figure 5, those two different dataset management options are illustrated. For explanation, the logical resource trees of the two modules are given to show their RESTful resources. The ‘set1′ dataset resource has a list of NGSI-LD entities that were discovered from the core module, while the ‘set2′ dataset instance contains a filter regarding the ‘type’ property. After dataset resource creations, when the application requests GET ‘/dataset/set1/data’ in HTTP, entity representations of ‘lot1′, ‘lot2′ and ‘lot3′ are returned, with attribute name modifications, if any were set. When GET ‘/dataset/set2/data’ is requested, the service module sends an entity discovery request to the core and sends back the NGSI-LD response with name changes, if needed. In the example, the ‘lot3′ instance, as an assumption, is created after the ‘set2′ dataset resource.

The data service module inherits the query parameter definition from the NGSI-LD API. Therefore, when a dataset is requested by an application with the query conditions, the service module uses the query parameter when it calls the NGSI-LD API to the core module.

**Figure 6:**
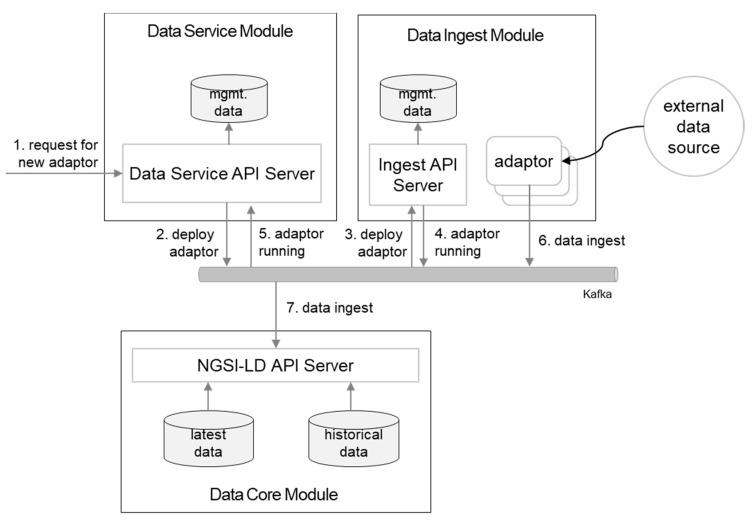
Dynamic ingest adaptor allocation for a new dataset.

When a provider does not have data in the data core but the data are located in an external system, the provider can request to allocate a new adaptor in the ingest module. Then, the new data become populated and shared with other stakeholders through the data marketplace. Figure 5 elaborates the procedures with request and data flows. Adaptor allocation requests and confirm messages are delivered over Kafka. Ae event-driven architecture style has been utilized for this purpose, and designated events are emitted upon a new adaptor deployment request and provide confirmation when it is up-and-running. When a new adaptor runs, that means a new data flow is active and other modules in the future can receive the event, which has detailed information of the flow, and consume the data feed. This is illustrated in Figure 6.

The access control mechanism involved in the data service module works as follows:

1. A data owner yields access management to the data service;
2. A data consumer makes a purchase on the marketplace portal, and it is recorded as a resource on the data service module;
3. Based on a dataset purchase records, the data service module grants access to the consumer who made the purchase.

As supported by the security module, each City Data Hub user has a JSON Web Token (JWT) [36], and the identity in the token is used to record the consumer’s identifier during the purchase. When there is a retrieval request for a dataset, the API server checks the requester’s identity with the ID in the token. If there is no matching ID among purchase information, the requests get rejected with an access denied error code.

### 4.4. Data Analytics

The data analytics module provides a toolbox which helps to obtain insights and solve problems with data in the platform. The City Data Hub design principle, to use standard data models, is also applied here. NGSI-LD-compatible data which are ingested to the data core module are used to train and build a prediction model. Then, the model is executed with new batch data input for inferencing. The prediction output as the result of the inferences can be used to update NGSI-LD entity instances with the NGSI-LD API.

Figure 6 extends Figure 3 to illustrate the interworking between the analytics and the data core modules. The data instances which are successfully stored into the data core are accumulated on the Hadoop Distributed File System (HDFS). There was the assumption that, later, the size of data that we deal with would be in petabyte scale, so big data analysis on a traditional RDBMS is not efficient. Instead, a framework based on a Hadoop ecosystem was selected, and this is the reason that the analytics module uses data on the HDFS rather than the RDBMS running in the core.

**Figure 7:**
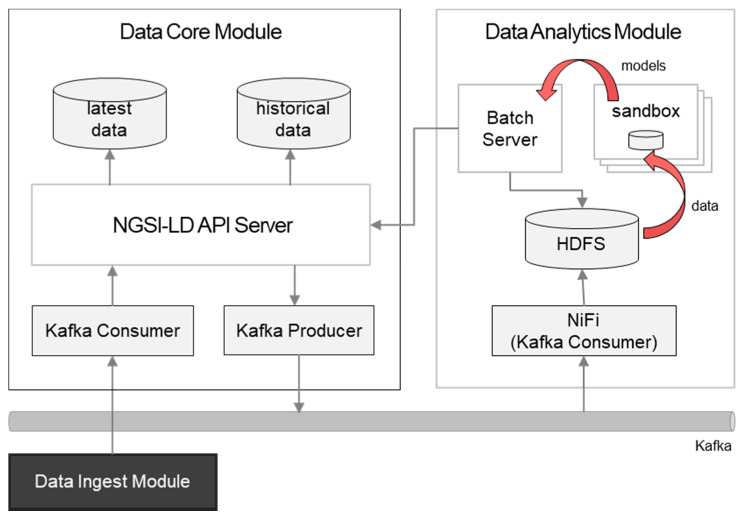
Interworking between the data core and analytics modules.

As the analytics module Extract, Transform, Load (ETL) tool, NiFi [37] consumes data from the Kafka message queue and creates data marts. After instance creation, the data ingest module normally sends update requests (e.g., number of available parking spots per parking event) targeting the existing instances. This update fragment consumed by the core module, after the successful update operation by the core API server, becomes a full representation when it sends Kafka messages with different topics. For data analysis, attributes that are not updated are also required so that the full entity instances per update become aggregated with timestamps, and they are used to create data marts based on use cases. Figure 7 describes this data flow.

From a user’s perspective, a data scientist creates machine learning models in the given data sandbox and, when they are tested, requests them to be used on the batch server to the analytics module admin. Only the granted models can run on the batch server since there can be NGSI-LD entity instance creations or updates on the core module.

Therefore, the first step for the data scientist is to create a sandbox for data pre-processing, models training and testing. Not just chunks of data but also other analytics tools are provided in the sandbox, including Hue [38] and Apache Hive [39]. In the sandbox create request, the user can choose or create a template that defines specific NGSI-LD entity types and periods (i.e., begin time and end time) of the sample data for the sandbox.

With the template, the sandbox is created on a Virtual Machine (VM) so that it can be used to compose a model. Currently, the scikit-learn library [40] in Python with its machine learning algorithms (e.g., RandomForestRegressor) is supported by the analytics module, and later, other libraries can be added.

To request model deployment on the batch server, detailed information to run models, such as model ID, sandbox ID, NiFi flow template ID and target NGSI-LD entity ID and Property name, are configured and delivered to the module admin.

## 5. Proof-of-Concept (PoC)

### 5.1. Parking Availability Prediction Service

This parking proof-of-concept (PoC) service was designed to demonstrate how different domain data in the City Data Hub can be used in terms of data mash-up and analytics. There has been much research on smart parking since parking has been considered a common traffic problem worldwide. Along with parking status information (e.g., parking spot occupancy), other types of data, such as weather forecasts, can be used for recommendation services [41].

We used three different data sources to develop the service and derived six data models from them [42]. Table 4 lists defined NGSI-LD entity types. To associate different data (e.g., parking lot availability and weather forecasts), common criteria were needed in the spatial and temporal domains. Therefore, the ‘address’ property for postal address [43] and ‘location’ for geo-location are included in all of the entity models as common properties. Those location-related properties as well as NGSI-LD standard timestamp properties (e.g., observedAt) were used to create the data mart by the analytics module.

**Figure 8:**
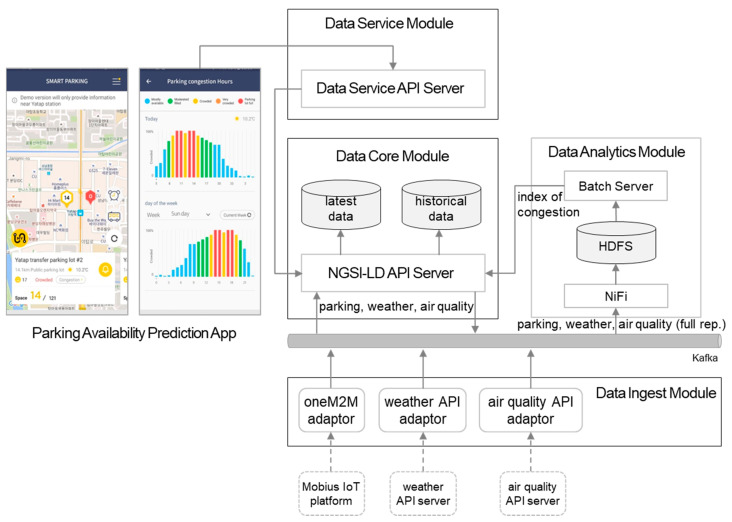
Parking availability prediction system.

The architecture and data flow for this service implementation are shown in Figure 8. Three adaptors for the three different APIs are used. Parking data were collected in public parking lots in Seongnam city into a oneM2M standard compatible platform, Mobius [46]. Other data pertaining to the air quality and weather were collected from the open APIs, so each API adaptor was deployed in the ingest module.

When data are stored into the core, they are provided to the application via the service module APIs. Furthermore, as illustrated in Section 4.4, data in full representation are accumulated into the analytic module. When periodical batch input data are served, the parking estimation machine learning model produces predicted congestion index values (e.g., hourly) and it is updated into an instance of the OffStreetParking entity.

### 5.2. COVID-19 EISS

**Figure 9:**
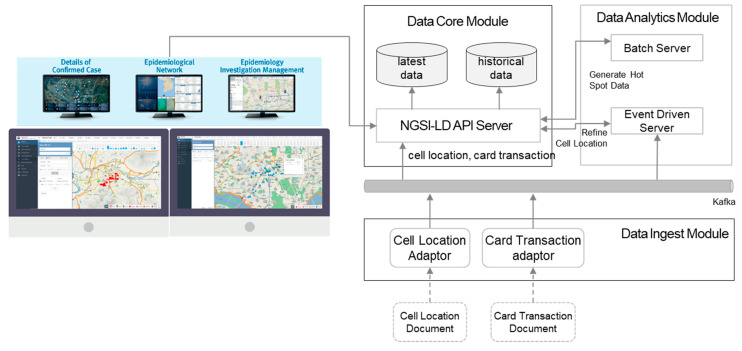
COVID-19 Epidemiological Investigation Supporting System (EISS) system.

In March 2020, due to the COVID-19 outbreak in South Korea, the Epidemiological Investigation Supporting System (EISS) was launched based on the City Data Hub [47]. The EISS, which is depicted in Figure 9, is basically the City Data Hub integrated with the investigation supporting service. When Excel file data are provided from mobile network operators or credit card companies, the file data are validated and ingested into the data core via Kafka. The number of records in an operator-provided location file per person and day is about 30 to 60 thousand. In one request, a number of people for several days are requested by users (e.g., investigators), and this is the moment when Kafka works better than HTTP for massive input.

Location data from cell towers of operator networks need to be filtered for duplicated data and outliers. When data pushes for all the records in the file are completed, a trigger event is emitted to perform data filtering. The filtered data, which are the processed data by the notion in Section 4.2.3, are stored in a different entity type. As the EISS feature, infection hot spots are analyzed by the analytics module and this is done periodically simply because it takes time.

Considering the situation back in early 2020, only three weeks were given by the Korea Center for Disease Control and Prevention (KCDC) to launch the service. This was the reason for choosing the file-based data ingest, which inevitably involves human intervention due to security regulations on those files inside the data provider systems. Towards the end of 2020, it will be replaced by API interworking, without any human involvement, between the EISS and the operator systems.

This system is under enhancement for other data analysis features, interworking with other data sources and with API interworking replacing file upload and extraction from the operators so that it can be used for other infectious diseases later.

## 6. Remaining Works

As depicted in the NGSI-LD interface specification [16], and as expected to be required in the near future with further adoptions, the platform federation architecture needs to be elaborated for the City Data Hub. On a smaller scale, for example, a city can deploy two types of the data hub. One is open for public, where any citizen can provide and consume data. The other is only available for special stakeholders, such as municipality agents, due to data governance and privacy. Between the two, a federation between the two hubs needs to be deployed. Furthermore, in the bigger picture, an inter-city data hub federation would be needed, too. Many data hubs can be running and there may be the need to manage distributed data by one.

Other types of data analytics are under development. Spatial data analytics from the COVID-19 EISS is being integrated and real-time stream data analytics are being implemented. Once those are done, different types of data analytics can be provided as a City Data Hub-provided common service.

Exploiting the linked data supported by the NGSI-LD in the data core, the semantics module has been also enhanced. Fundamental features such as a semantic annotator to populate RDF (Resource Definition Framework) triple stores were implemented. For the annotator, smart city common ontology was defined and extensions were also made for domain-specific ontologies (i.e., the parking service PoC) [48]. In addition to the traditional approach, in future plans, such as including a semantic validator and reasoner, we aim to build a graph database adaptor for NGSI-LD. There have been efforts to implement the NGSI-LD APIs on the Neo4j database [49], but applications consume information with NGSI-LD APIs. Our plan is to obtain data from the NGSI-LD and synchronize information into a graph database (e.g., Arango DB) to provide graph traversal features which are not supported by the NGSI-LD RESTful APIs.

## 7. Conclusions

In this paper, we presented the proven outcome, the City Data Hub, from the ongoing Korean smart city project. To have interoperable and replicable smart city platforms with different infrastructures and services, we chose well-known mechanisms from the relevant research and deployment experiences: (1) standard APIs, (2) common data models and (3) data marketplaces.

The major contribution from our work is that a data-driven smart city platform has been designed and implemented with NGSI-LD standard APIs and compatible data models. Internal functional modules (e.g., data ingest, data core and data analytics modules) of the City Data Hub interwork using NGSI-LD, and external smart city services also receive data with the interfaces and data models. We have described how data are collected from IoT platforms and how the modules exchange the data over the adopted standards.

There are many IoT systems that belong to different domains and provide heterogeneous data to smart city data platforms. Since the linked data concept is supported by NGSI-LD in the City Data Hub, cross-cutting applications of the City Data Hub should be able to exploit the full potential of data linkage.

Two PoC implementations demonstrated how the City Data Hub can be used for problem solving in smart cities utilizing data and APIs. Overseas deployment, including cross-city interworking, is planned; therefore, data governance, privacy and regulations can be drawn and considered for future enhancement.

Like the FIWARE ecosystem approach, systems composed of standard APIs enabled GEs [50]. The ecosystem of the City Data Hub can be extended with start-ups and research institutes contributing new modules, such as atomic services, in the SynchroniCity framework. Furthermore, education and consulting businesses can be followed as new systems deploy the data hub.

Existing modules and upcoming ones should be certified for commercial markets. The testing system for the core module standard APIs has been verified within the project and it is going to be applied to other modules. There will be a need for product profiles for certification so that each product can choose modules or features that they implement from the data hub specifications.

In terms of openness, as mentioned, reference implementations from the project are going to be provided as open sources as well as the specification publication. Open-source solutions tend to survive and evolve better than proprietary ones thanks to contributions from others, and this applies to smart cities [51]. This open-source strategy should boost the formation and broadening of the City Data Hub ecosystem and possibly collaborate with other open-source communities.

## Figures and Tables

**Table 1:** Dynamic schema description notation.

Property	Multiplicity	Data Type	Description
entityType	1	String	‘type’ value of NGSI-LD entity
version	1	String	scheme version
description	1	String	scheme description
indexAttributeIds	0..N	String	attribute IDs for indexing
rootAttributes	0..N	Attribute ^1^	‘Property’ or ‘Relationship’ of NGSI-LD entity

^1^ Attribute data type is defined in Table 2 as complex data type.

**Table 2:** Definition of Attribute data type.

Child Property	Multiplicity	Data Type	Description
id	1	String	attribute ID
required	0..1	Boolean	attribute cardinality
type	1	String	‘Property’, ‘GeoProperty’, or ‘Relationship’
maxLength	0..1	String	max length of digits or max number of characters
valueType	1	String	E.g. ‘GeoJSON’, ‘String’, ‘ArrayString’, ‘Object’
valueEnum	0..N	String	list of allowed enumeration values
observedAt	0..1	Boolean	this is true when a property includes ‘observedAt’ timestamp
objectKeys	0..N	ObjectKey ^1^	when valueType is ‘object’, its key IDs and value types are defined
childAttributes	0..N	Attribute	child ‘Property’ or ‘Relationship’ of NGSI-LD entity

^1^ ObjectKey data type is defined in Table 3 as complex data type.

**Table 3:** Definition of ObjectKey data type.

Child Property	Multiplicity	Data Type	Description
id	1	String	attribute ID
required	1	Boolean	attribute cardinality
maxLength	0..1	String	max length of digits or max number of characters
valueType	1	String	‘String’, ‘ArrayString’, ‘Integer’, ‘ArrayInteger’, ‘Double’, or ‘ArrayDouble’

**Table 4:** A list of data models for parking service.

Entity Type	Entity Specific Property	Reference
OffStreetParking	availableSpotNumber, congestionIndexPrediction	[13,43]
ParkingSpot	occupancy	[13,43]
AirQualityObserved	airQualityIndexObservation, indexRef	[44]
AirQualityForecast	airQualityIndexPrediction, indexRef	[44]
WeatherObserved	weatherObservation	[45]
WeatherForecast	weatherPrediction	[45]
